# A service-oriented architecture for integrating the modeling and formal verification of genetic regulatory networks

**DOI:** 10.1186/1471-2105-10-450

**Published:** 2009-12-30

**Authors:** Pedro T Monteiro, Estelle Dumas, Bruno Besson, Radu Mateescu, Michel Page, Ana T Freitas, Hidde de Jong

**Affiliations:** 1 INRIA Grenoble - Rhône-Alpes, 655 Avenue de l'Europe, Montbonnot, 38334 St Ismier Cedex, France; 2 INESC-ID/IST, Rua Alves Redol 9, 1000-029 Lisbon, Portugal; 3 Institut d'Administration des Entreprises, Université Pierre Mendès France, Grenoble, France

## Abstract

**Background:**

The study of biological networks has led to the development of increasingly large and detailed models. Computer tools are essential for the simulation of the dynamical behavior of the networks from the model. However, as the size of the models grows, it becomes infeasible to manually verify the predictions against experimental data or identify interesting features in a large number of simulation traces. Formal verification based on temporal logic and model checking provides promising methods to automate and scale the analysis of the models. However, a framework that tightly integrates modeling and simulation tools with model checkers is currently missing, on both the conceptual and the implementational level.

**Results:**

We have developed a generic and modular web service, based on a service-oriented architecture, for integrating the modeling and formal verification of genetic regulatory networks. The architecture has been implemented in the context of the qualitative modeling and simulation tool GNA and the model checkers NUSMV and CADP. GNA has been extended with a verification module for the specification and checking of biological properties. The verification module also allows the display and visual inspection of the verification results.

**Conclusions:**

The practical use of the proposed web service is illustrated by means of a scenario involving the analysis of a qualitative model of the carbon starvation response in *E. coli*. The service-oriented architecture allows modelers to define the model and proceed with the specification and formal verification of the biological properties by means of a unified graphical user interface. This guarantees a transparent access to formal verification technology for modelers of genetic regulatory networks.

## Background

The study of genetic regulatory networks, as well as other biological networks, has led to the development of increasingly large and detailed models [1]. The models consist of dozens or even hundreds of variables describing the molecular species involved in a variety of intracellular processes [2-7]. Computer tools are essential for the simulation of the dynamical behavior of the networks from the models, for instance when predicting the response of the system to an external perturbation. However, as the size of the models grows, it becomes infeasible to manually verify the predictions against experimental data or identify interesting features in dozens of simulation traces. This calls for the use of automated and scalable methods that help the modeler with the identification and verification of interesting dynamical properties of the network. The field of formal verification provides promising methods to prove or disprove specified properties of a system. These methods proceed by an exploration of all possible behaviors of the system, following two main approaches: logic inference, based on the use of axioms and proof rules [8], and model checking, based on an automatic and exhaustive search of the state space [9]. In this paper, we focus on the model checking approach. The basic idea underlying model checking is to specify dynamical properties of interest as statements in temporal logic, and to use model-checking algorithms to automatically and efficiently verify whether the properties are satisfied or not by the model [9]. In recent years, several examples of the application of model checking to the analysis of biological regulatory networks have been published in the literature (*e.g.*, [10-21]).

According to our experience, there are currently two major obstacles that prevent modelers in systems biology from drawing maximal benefit from formal verification tools. First, the formulation of biological questions in temporal logic and the interpretation of the verification results is far from obvious, especially for non-expert users who are not used to this kind of reasoning. Second, most of the existing modeling and simulation tools are not capable of applying model-checking techniques in a transparent way. In particular, they do not hide from the user the technical details of the installation of the model checker, the export in a suitable format of the model and the query, the call of the model checker, and the import of the results produced by the model checker (the true/false verdict and witnesses/counterexamples). In other words, what is missing is a framework that tightly integrates modeling and simulation tools with formal verification tools, on both the conceptual and the implementational level.

In order to address these issues, we propose a service-oriented architecture (SOA) [22] for the integrated modeling and formal verification of genetic regulatory networks, which reuses existing technology as much as possible. The architecture connects modeling and simulation clients to a formal verification server, *via *an intermediate request manager. In particular, the client can perform verification requests through the web, which the request manager dispatches to an appropriate formal verification server. When the formal verification server has answered the request, the results are sent back to the modeling and simulation client for display and further analysis in the graphical user interface of the tool. The interactions of the client with the remote web server are handled by a verification module assisting the specification of biological queries through a property editor, either by directly choosing the appropriate temporal logic operators or by using a tailored set of query patterns [23].

The architecture is generic and modular, but we develop it here in the context of one particular modeling and simulation tool (GNA [24]) and two different model checkers (NUSMV [25] and CADP [26]). A first generalization of the work presented here would be to integrate other formal verification tools into the architecture. This possibility is anticipated through the use of a plugin system, where each plugin contains all data transformations and operations specific to a particular formal verification tool. This simplifies the integration of a new tool to the creation of the corresponding plugin. A second generalization would be to extend the service-oriented architecture to other modeling and simulation tools. A variety of tools have been used in combination with model checkers, such as GINSIM [27], INA [28], BIOCHAM [29], GNA [24] or ROVERGENE [14], based on formalisms like Boolean and other logical models [30-32], Petri nets [21,33,34] or ordinary differential equations [35,36]. In order to integrate new modeling and simulation tools into the architecture, they each have to be equipped with a verification module that interacts with the request manager, sending verification requests and receiving answers and diagnostics, as well as a plugin system to define the contents of the messages.

In the next section, we describe the service-oriented architecture and its components in detail and we motivate the most important implementation choices. The practical use of the architecture is then illustrated by means of a scenario involving the analysis of a qualitative model of the carbon starvation response in *E. coli*. The model describes a network of key global regulators of the bacterium, responsible for the control of the expression of a large number of stress response genes [37,38]. We trace the different steps from the formulation of a temporal logic query to the visualization and interpretation of the verification results. The discussion summarizes our contributions and places it in the context of related work.

## Implementation

**Figure 1:**
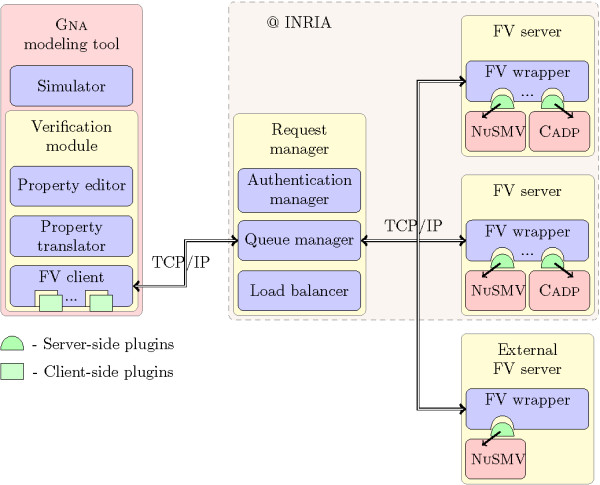
**Service-oriented architecture**. Service-oriented architecture for the integration of tools for the modeling and simulation of genetic regulatory networks with formal verification (FV) tools. In particular, the architecture has been implemented for the connection of GNA with the model checkers NUSMV and CADP. GNA is extended with a verification module responsible for the transformation of the model and properties into a format specific to a formal verification tool, and for the communication with the other components of the service-oriented architecture.

In this section, we describe the overall architecture of the system (Figure 1), with a step-by-step description of its components: the modeling and simulation tool with its verification module, the request manager, and the formal verification server. These three components have been implemented in Java 1.5 and their web-service interface is based on Apache Axis 1.3 http://ws.apache.org/axis/.

The implementation followed two main principles: a service-oriented architecture and the use of plugins. A service-oriented architecture is particularly well suited for our purpose. The formal verification service is remotely executed through the web and is implemented using standard protocols and languages like TCP/IP, SOAP and XML. A GNA user wishing to perform a verification request does not need to install a model checker or other formal verification tool locally on his or her machine. The use of plugins provides a flexible and extensible way to abstract a particular formal verification tool. It allows one to apply the tool without worrying about the details of its implementation.

### Modeling and simulation tool

The service-oriented architecture is accessible for users of version 7.0 of the qualitative modeling and simulation tool Genetic Network Analyzer (GNA), available as described at the end of the paper. GNA uses a class of piecewise-linear (PL) differential equations, providing a coarse-grained picture of the dynamics of genetic regulatory networks [39]. The models associate a protein concentration variable to each of the genes in the network, and capture the switch-like character of gene regulation by means of step functions that change their value at threshold concentrations of regulatory proteins. The advantage of using PL models is that the qualitative dynamics of the high-dimensional systems are relatively simple to analyze, using inequality constraints on the parameters rather than exact numerical values [13,40]. This makes the PL models a valuable tool for the analysis of genetic regulatory networks in the absence of quantitative information on the parameter values. The graphical user interface of GNA supports the modeler in building step-by-step a PL model of the network under study (see the tutorial available from the GNA web site for details and examples).

GNA computes discrete abstractions of the continuous dynamics of the PL models, resulting in a finite-state transition system (FSTS) defined as a quintuple Σ = ⟨ *S, S*_0_, *AP, L, T*⟩ [9]. *S *is a set of states, where each state corresponds to a hyperrectangular region in the concentration space, defined by the thresholds of the concentration variables. *S*_0 _⊆ *S *is the set of initial states. *AP *is a set of atomic propositions, related to the states by means of a labeling function *L*: *S *→ 2^*AP*^. The labeling function determines which atomic propositions are satisfied in a particular state *s *∈ *S*. The atomic propositions concern among other things, the thresholds bounding the concentration variables, the signs of the derivatives of the concentration variables, and indicate if the state is a steady state. *T *represents the set of transitions between the states, where each transition corresponds to a solution trajectory entering one region from another [40]. GNA allows the user to visualize the FSTS, *i.e. *to display the corresponding state transition graph, and analyze the atomic propositions characterizing the states. For large graphs visual inspection quickly becomes infeasible and formal verification tools are needed. Previous versions of GNA supported the export of the FSTS to text files accepted by several model checkers [13,41]. Version 7.0 extends GNA with a verification module that integrates the tool into the service-oriented architecture.

### Verification module

The verification module consists of three components: a pattern-based property editor, a property translator, and a formal verification client (Figure 1).

#### Pattern-based property editor and translator

The problem of posing relevant and interesting questions is critical in modeling in general, but even more so in the context of applying formal verification methods, due to the fact that is not easy for non-experts to formulate queries in temporal logic. The pattern-based property editor is a user interface that allows the specification of biologically-relevant properties in the form of temporal logic formulas. The specification of properties can be achieved in two distinct ways: for common biological properties through the use of a pattern system, and for more specific or complex properties through the use of a text editor of temporal logic formulas.

Patterns are high-level query templates that formulate recurring questions in the analysis of regulatory networks using a domain-specific language [42] rather than temporal logic. They were originally introduced in the formal verification field [43] and recently adapted for use in systems biology [23]. From a study of the literature on the modeling of biological regulatory networks, it was found that most of the questions asked by experts can be reduced to a set of four patterns concerning the: *occurrence/exclusion*, *consequence*, *sequence *and *invariance *of events. Notice that these patterns are classes of properties sufficiently generic to be applicable in a variety of systems biology models, and that the aim of these patterns is not to cover all possible questions the modeler can think of, but rather to simplify the formulation of the most frequent or otherwise important ones.

**Figure 2:**
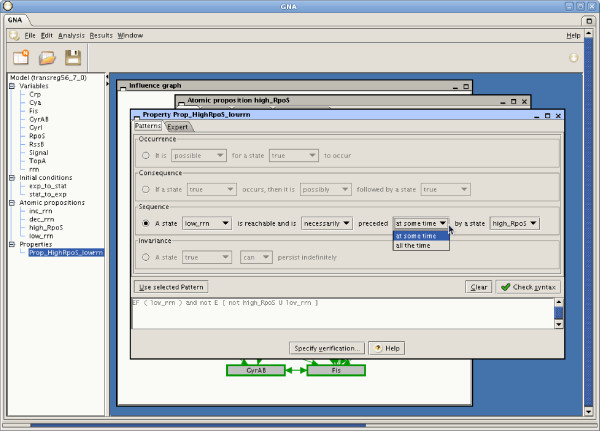
**Pattern-based property editor**. Graphical user interface for the specification of biological properties. The modeler can use a pattern-based property editor for frequently-asked questions, and a text editor for the specification of more complex biological properties (expert mode).

Figure 2 shows the pattern-based property editor of GNA. It presents the four different types of patterns as templates to be completed by the user. The completion of the templates requires the modeler to have previously defined atomic propositions, each of which describes characteristics of a state of the network, such as an increasing or decreasing protein concentration, a steady state, or a protein concentration above a certain threshold. When a pattern has been specified, it is automatically translated into Computation Tree Logic (CTL) [9]. The pattern-based property editor and translator are also available as a stand-alone Java application (see Availability and requirements below). An application programming interface (API) is provided, so that the patterns can be integrated into other modeling tools that wish to implement the encoding of biological properties into temporal logic formulas (CTL, CTRL and μ-calculus are currently supported).

More complex biological properties can be directly specified in the Computation Tree Regular Logic (CTRL) language [44]. CTRL extends CTL with regular expressions and fairness operators, which favors the expression of properties like multistability or oscillations, and endows the logic with a user-friendly syntax. The text editor allows the modeler to specify any temporal logic formula by freely combining the set of CTL and CTRL operators with propositional logic operators and the user-defined atomic propositions. The temporal logic properties can be stored for later use with the GNA model in a single project file.

#### Formal verification client and client-side plugins

The formal verification client is the component that enables GNA to communicate with the request manager. It thus gives the user an easy access to the formal verification technology without having to locally install a tool or worrying about how to get it to work. To perform a verification request, the modeler needs to choose which tool to use, which property to verify, *etc. *These choices may be guided by the estimation of the model size (*e.g.*, for large regulatory networks containing dozens of genes, symbolic model checking is likely to scale up better than explicit-state model checking) or by the nature of the properties to be verified (*e.g.*, linear-time or branching-time, with/without regular expressions, *etc.*). The FSTS on which the property is to be verified can be defined explicitly or implicitly. In the former case, the FSTS is completely generated by the simulation module of GNA, while in the latter case it is given by the set of initial states and a function that computes the successors of any given state. The formal verification client performs a request by sending the implicit or explicit description of the FSTS through the web and waiting for the result. The implicit definition has the advantage of considerably reducing the size of the specification of the FSTS, and thus limiting the size of the files transmitted and the response delays. This may be critical for large FSTSs. The verification result is composed of a true (false) verdict supported by a witness (counterexample). The witness or counterexample consists of a sequence of states in the FSTS, displayed in the graphical user interface of GNA.

In order to make the verification module of the modeling tool independent of a specific formal verification tool, we have developed a plugin system. Currently, a plugin for the model checker NUSMV is available, while a beta version for CADP has been completed. All data transformations specific to a particular model checker are taken in charge by the corresponding plugin, thus leaving the service-oriented architecture free to manage generic verification requests. Each plugin has a client-side and a server-side (Figure 1). The client-side plugin has the responsibility of translating the FSTS and the property into a format accepted by the corresponding formal verification tool, while the server-side plugin is in charge of receiving the translated FSTS and property, feeding them into the formal verification tool executable, and parsing the results returned by the tool.

At the present time, the model checkers integrated in the architecture are invoked using the default parameters. More elaborate choices could be partially automated by incorporating into the plugin some knowledge of the verification method and the underlying algorithms.

### Request manager

The request manager is a component of the service-oriented architecture with a public address http://java1.inrialpes.fr, acting as an intermediary service that ensures the communication between all the modeling tools and formal verification servers.

#### Queue and authentication manager

In order to keep track of the state of all verification requests and the available formal verification servers, a queue and authentication manager has been implemented. Upon each verification request the authentication manager, together with the server-side plugin, checks for the credentials of the request. If successful, the queue manager registers the request in the queue, checks for an available formal verification server, and hands over the request. The queue manager will continue to poll the formal verification server for a response until one of three events happens: the verification has completed, the user has aborted the verification request, or a timeout has occurred. The verification result (verdict and counterexample) is then returned to the user.

To ensure the service security, each authenticated request registered in the queue, generates an Universally Unique Identifier (UUID) that is returned to the client, so that only this client is able to retrieve the verification result. Furthermore, when the result is retrieved, both the request manager and the formal verification server that handled the request delete the model and temporal logic formula, leaving no traces of the request in the server.

#### Load balancer

The service-oriented architecture has been designed to support several formal verification servers. The address of every server, as well as all the model checkers and other formal verification tools types locally installed on each of the servers, are registered in the request manager. Upon a verification request, the load balancer chooses an idle formal verification server with the required tool and server-side plugin installed. When all formal verification servers are busy, the load balancer waits until one becomes idle.

### Formal verification server

A formal verification server has the responsibility of verifying properties submitted by the request manager. One or several formal verification tools can be installed on a server provided that the corresponding server-side plugins are also installed on this server.

#### Formal verification server and server-side plugins

The formal verification server contains the web-service interface, which is responsible for receiving the requests from the request manager, the choice of the corresponding server-side plugin, and the construction of the verification result to be returned.

Each plugin specific to a formal verification tool has an authentication module which responds to the authentication requests made by the request manager. In addition, upon a verification request, the plugin pre-processes the model description and the property in order to transform them into the format accepted by the formal verification tool, and calls the latter with the appropriate parameters. When the formal verification tool finishes the verification of the request, it produces the verdict as well as the corresponding witness (or counterexample).

Since this witness has a format specific to a particular formal verification tool, it is up to the plugin to parse the results and perform the necessary data transformations to a common format that is sent back to the modeling tool. The required transformations depend on whether the request involves a FSTS of an implicit or explicit type. In the explicit case, the witness is simply a subgraph of the FSTS sent to the formal verification tool, whereas in the implicit case the state information needs to be reconstructed from the output of the tool.

### Integration of new formal verification tools

An important advantage of the chosen architecture, which delegates all operations that are specific to a particular formal verification tool to plugins, is that it allows for the flexible integration of new tools. Two plugins have been developed until now: one for NUSMV (released with the GNA distribution) and one for CADP (beta version completed). Developers wanting to develop plugins for different model checkers or other formal verification tools can do so through the following main steps: the development of a client-side plugin, the development of a server-side plugin, and the installation of the server-side plugin on a server on which the new tool is running.

The client-side plugin takes a .jar file that must be placed in the *plugins *directory of GNA, allowing the modeling tool to export the FSTS to a file that can be read by the new formal verification tool. GNA dynamically recognizes the available client-side plugins, using the Java Plugin Framework technology. The development of a server-side plugin results in a Java class that needs to be copied in an appropriate directory of the wrapper on the formal verification server. The latter server must register its web service connection parameters in the request manager, so as to enable the latter to dispatch the requests to the correct formal verification server. More detailed information on the development of plugins can be obtained by contacting the authors directly.

## Results

**Figure 3:**
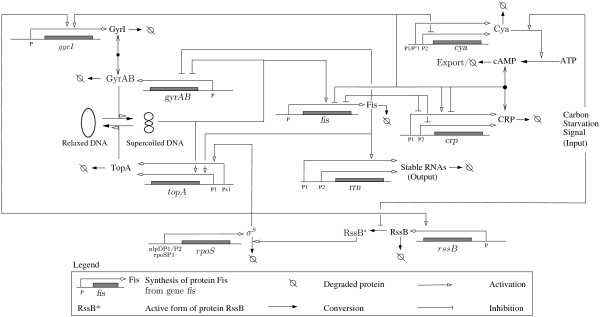
**Carbon starvation response network in E. coli**. Network of key genes, proteins and regulatory interactions involved in the carbon starvation response network in *E. coli *[37,38].

In order to illustrate the use of the web service, we present a scenario using a PL model of the network of global regulators controlling the carbon starvation response in the enterobacterium *Escherichia coli*. In order to survive, *E. coli *cells constantly have to adapt their functioning to the availability of carbon sources, essential for growth. The adaptation involves multiple levels of regulation, from metabolic fluxes and enzyme activity to gene regulation [45-47]. In this example, we focus in particular on the role of the global regulators of transcription, such as CRP, Fis, DNA supercoiling, and RpoS. These global regulators form the backbone of the network coordinating the long-term response of *E. coli *cells to starvation conditions (Figure 3). The PL model consists of 9 equations and more than 50 parameter inequalities that specify the qualitative dynamics of the system [38]. Below, we illustrate how the specification and verification of temporal logic properties can help the analysis of the role of RpoS in the dynamics of the system.

### Property specification procedure

RpoS or σ^*s *^is a sigma factor that allows cells to adapt to and survive under harmful conditions by expressing a variety of stress response genes [48]. Due to its key role in the cell, the concentration of RpoS is tightly regulated at the transcriptional, translational, and post-translational levels. It this section, we focus on the conditions of stability of the protein. While cells grow on a carbon source, RpoS is actively degraded through the protein RssB, which binds to RpoS and targets the factor to an intracellular protease (Figure 3). However, the depletion of the carbon source inactivates RssB, thus allowing RpoS to accumulate to a high concentration. Given the important role of RpoS for the survival of the cell, one may ask whether the entry into stationary phase upon carbon starvation is always preceded by the accumulation of RpoS in the cell.

The first step in answering this question using the formal verification module of GNA consists in identifying elements of the question that refer to the state of the biological system and in stating these as atomic propositions. We represent the entry into stationary phase of the system by a low level of stable RNAs encoded by the *rrn *operons. This is motivated by the fact that stationary-phase cells do not need high levels of stable RNAs, contrary to what is required by the high translational activity in exponential phase. These characteristics are specified using the property editor, where we create an atomic proposition named *low_rrn *(Additional file 1), restricting the concentration values for the variable *rrn *to those below its (single) threshold. We also introduce an atomic proposition *high_RpoS*, representing the accumulation of RpoS to a value above its threshold *t_RpoS *(Additional file 2).

The second step is the formulation of the biological property using the pattern-based property editor and translator. We choose the *sequence *pattern to account for the temporal ordering of the two states: stationary phase and high expression of RpoS. The sequence pattern is instantiated by selecting the previously defined atomic propositions (Figure 2):

"A state | *low_rrn *| is reachable and is | *necessarily *| preceded | *at some time *| by a state | *high_RpoS*".

Once the pattern is fully instantiated, it is automatically translated into the corresponding CTL formula: EF (*low_rrn*)∧¬E (¬*high_RpoS* ⋃ *low_rrn*).

### Property verification procedure

After the specification of the property, one passes to the verification stage. For this step, the verification request must be configured in the verification window (Additional file 3). First, we choose the name and version of the model checker plugin to be used (version 1.0 of the NUSMV implicit plugin was used in the example). Second, we specify the initial conditions. The resulting implicit FSTS represents the transition from exponential phase to stationary phase, starting from initial conditions corresponding to carbon depletion. We then run the verification request, which is treated by the service-oriented architecture as described in the previous section.

**Figure 4:**
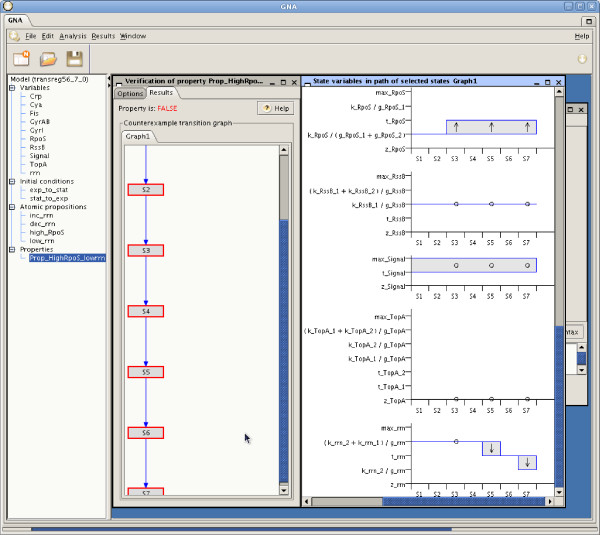
**Verification result**. Result of the verification of the biological property specified in Figure 3, consisting of a false verdict and the corresponding counterexample composed of a subgraph of the FSTS (see left panel). The qualitative evolution of the concentration variables of the selected states of the counterexample is visualized (see right panel).

In response to the query the model checker returns false after 4 seconds. It means that the entry into stationary phase is not always preceded by the accumulation of RpoS in the cell. The counterexample is presented to the user as shown in the left panel of Figure 4. It consists of a subgraph of the initial FSTS, starting from the specified initial state and ending in a state where the property fails. By selecting a path in this subgraph, GNA allows the qualitative changes in the concentration of all the variables to be displayed (right panel of Figure 4). Looking at the evolution of the variables we immediately observe that there is (at least) one sequence of states leading to a low expression level of the *rrn *operons without having previously passed through a state with a high concentration of RpoS. This illustrates the negative verification result, and witnesses that the downregulation of the stable RNAs does not require the previous accumulation of RpoS.

### Another verification example

Continuing with the previous analysis, one may want to look into the role of RpoS in the control of DNA supercoiling during growth-phase transitions. The DNA supercoiling level is regulated by the gyrase GyrAB, which supercoils the DNA structure, and by the topoisomerase TopA, which relaxes it.

In order to know whether *topA *is expressed in response to the carbon source availability, we create an atomic proposition named *low_topA *representing the low expression of *topA*, and we choose the following *invariance *pattern to check if the absence of *topA *expression persists indefinitely:

"A state|*low_topA*|can|persist indefinitely".

The corresponding translation of this pattern is the following CTL formula: EG (*low_topA*).

Following the previously described verification procedure, the formal verification server returns false after 3 seconds, and the counterexample shows that expression of *topA *is stimulated at the entry into stationary phase, under the influence of RpoS. Indeed, following carbon starvation, the protein RssB is inactivated, which leads to the accumulation of RpoS at high levels. RpoS in turn activates the *topA *promoter. Complex properties like the existence of oscillations can also be verified. If the property holds, the verification module will present the corresponding lasso-shaped witness (Additional file 4) for visual inspection.

## Discussion

In this paper, we have proposed a generic and modular service-oriented architecture to integrate the modeling of genetic regulatory networks with existing formal verification tools. Currently, the service-oriented architecture connects the GNA modeling tool, extended with a formal verification module, with the NUSMV and CADP model checkers. We have given a detailed description of the existing components and motivated our implementation decisions. Additionally, we have illustrated the use of this architecture with the analysis of the complex network of global regulators involved in the carbon starvation response in *E. coli*. GNA is freely available for non-profit academic research, while the main component of the formal verification module, the pattern-based property editor and translator, is also available separately (see Availability and requirements below).

Formal verification methods have historically been used for the verification of hardware and software systems. Some of the existing model checkers, such as PRISM [49] and NUSMV [25], have recently been applied to the verification of biological systems. PRISM verifies properties specified in Continuous time Stochastic Logic (CSL) and has been used to perform quantitative analysis [17] of the ERK intracellular signaling pathway model [50]. NUSMV has been used for the analysis of biological models like the carbon starvation response in *E. coli *[13], the cell-cycle control in *C. crescentus *[20], the mucus production in *P. aeruginosa *[15], and the mammalian cell-cycle control [18]. In most cases, the biological models are built using modeling tools that are not connected to model checkers. Some modeling tools like GINSIM [27] and previous versions of GNA [41] are capable of exporting the model in an implicit or explicit format accepted by the model checker and the entire analysis is carried out in the model-checking environment, without any feedback to the modeling tool. An exception is the modeling tool BIOCHAM [29], which integrates the model checker NUSMV and allows for a more flexible iterative modeling and verification approach.

## Conclusions

In this paper we carried further the integration of the modeling and formal verification of biological networks, by proposing a service-oriented architecture that presents several advantages. First of all, the proposed connection between modeling and verification tools is completely transparent for the modeler and platform-independent. It requires web access but this is becoming less and less of a constraint in the current age of pervasive internet use. Second, the web-service based integration of the tools coming from different domains makes it possible to exploit the strong points of each. On the modeling side, the graphical user interfaces present the properties to be verified and the verification results in a way accessible to the modeler. For instance, the specification of biological properties by means of query patterns [23] does not require prior knowledge of any specific temporal logic. On the verification side, the latest developments of state-of-the-art model checkers can be immediately integrated. Third, the plugin system provides a modular way to add new formal verification methods without having to develop a new version of the modeling tool. The upgrade to future releases of a formal verification tool can also be performed through a simple plugin update.

The architecture has been implemented in the context of GNA, but generalizations to other modeling and simulation tools is obviously possible and facilitated by the modular structure. The integration of such tools into the architecture requires them to implement a verification module responsible for the specification of biological properties, the call of plugins for specific formal verification tools and the exchange of verification requests with the request manager. However, this implementation work is facilitated by the availability of the pattern-based property editor as a stand-alone Java application. In addition, the development of new plugins for tools based on model formalisms that can be mapped to FSTSs, explicitly or implicitly, are conveniently designed after plugins already available for GNA.

Formal verification methods are promising tools for upscaling the analysis of genetic regulatory networks. The widespread adoption of these approaches has been hampered so far, by the difficulty for non-expert users to formulate appropriate questions in temporal logic, effectively use formal verification tools, and meaningfully interpret the results returned by the model checker. The modular infrastructure that we propose is capable of connecting modeling and formal verification tools. In combination with graphical user interfaces capable of presenting data in a form accessible to modelers, we expect this to lower the obstacles to the use of formal verification technology in biology.

## Availability and requirements

**Project name**: Genetic Network Analyzer 7.0 (including the NUSMV plugin)

Project home page: http://ibis.inrialpes.fr/article122.html

Operating system(s): Platform independent (Windows, Linux, MacOS)

Programming language: Java 1.5

License: GNA is distributed by Genostar http://www.genostar.com/. Free license for non-commercial academic users granted upon request on the GNA home page.

Any restrictions to use by non-academics: contact Genostar at info@genostar.com for conditions.

**Project name**: Procrustes: Pattern-based property editor

Project home page: http://ibis.inrialpes.fr/article938.html

Operating system(s): Platform independent (Windows, Linux, MacOS)

Programming language: Java 1.5

License: LGPL

## Authors' contributions

PTM designed and implemented the service-oriented architecture as well as the pattern editor and the NUSMV and CADP plugins, carried out the analysis of the *E. coli *example and drafted the manuscript. ED designed and implemented the service-oriented architecture and the CADP plugin. BB helped with the implementation of the service-oriented architecture. RM helped with the design of the pattern editor and the design and implementation of the CADP plugin. MP designed the service-oriented architecture, helped with the design and implementation of the NUSMV plugin, and helped to draft the manuscript. ATF helped with the design of the pattern editor and helped to draft the manuscript. HdJ helped with the design of the service-oriented architecture as well as the NUSMV and CADP plugins, helped with the analysis of the *E. coli *example and drafted the manuscript. All authors read and approved the final manuscript.

## Supplementary Material

Additional file 1 **Definition of the atomic proposition *low_rrn***. Atomic proposition specification window, where atomic propositions are defined in terms of restrictions applied to a state (*e.g.*, restrictions on concentration values, focal sets, derivatives, and other state descriptors). In this case, the value of the concentration is restricted to lie below the threshold *t_rrn*. Click here for file

Additional file 2 **Definition of the atomic proposition *high_RpoS***. Atomic proposition specification window, where atomic propositions are defined in terms of restrictions applied to a state (*e.g.*, restrictions on concentration values, focal sets, derivatives, and other state descriptors). In this case, the value of the concentration is restricted to lie above the threshold *t_RpoS*. Click here for file

Additional file 3 **Verification options window**. Configuration of a verification request by specifying the model checker plugin to be used and, if the plugin supports an implicit representation of the FSTS, the initial conditions for the qualitative simulation of the network. Click here for file

Additional file 4 **Verification result**. Results of the verification of a complex biological property, composed of a verdict (true) and the corresponding witness. The latter consists of a sequence of states containing a cycle (see left panel). The value of the concentration of the variables in the selected states is shown, presenting an oscillation of the concentration of the variable *Fis *(see right panel). Click here for file
