# Supplementary material for: A service-oriented architecture for integrating the modeling and formal verification of genetic regulatory networks
Source: BMC Bioinformatics. 2009 Dec 30;10:450. doi: 10.1186/1471-2105-10-450 (PMC2813247; doi:10.1186/1471-2105-10-450)
Supplement: Additional file 1 — Definition of the atomic proposition low_rrn. Atomic proposition specification window, where atomic propositions are defined in terms of restrictions applied to a state (e.g., restrictions on concentration values, focal sets, derivatives, and other state descriptors). In this case, the value of the concentration is restricted to lie below the threshold t_rrn. [file 1471-2105-10-450-S1.PDF]

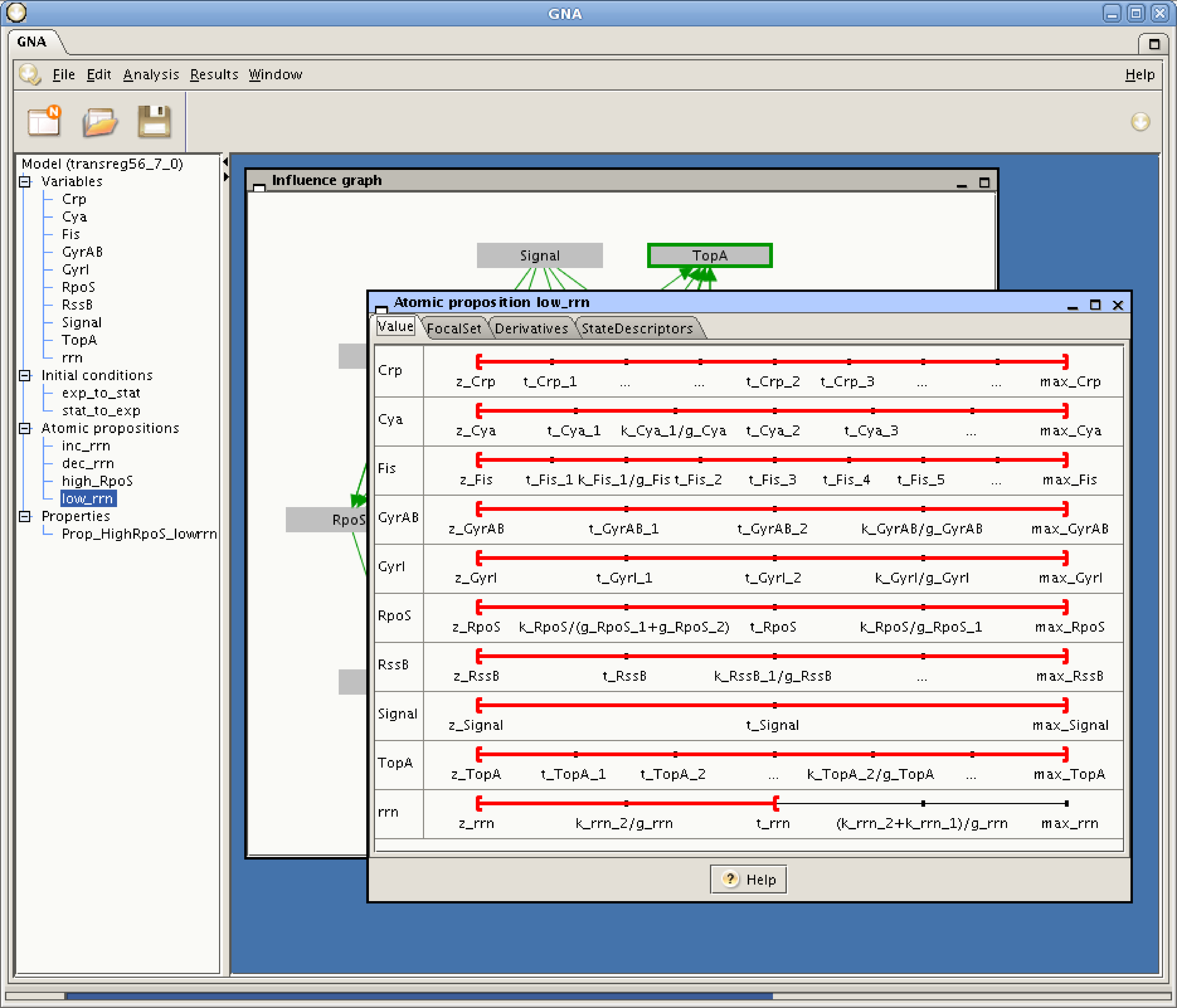
GNA
GNA
File Edit Analysis Results Window
Help
Model (transreg56_7_0)
Variables
Crp
Cya
Fis
GyrAB
GyrI
RpoS
RssB
Signal
TopA
rrn
Initial conditions
exp_to_stat
stat_to_exp
Atomic propositions
inc_rrn
dec_rrn
high_RpoS
low_rrn
Properties
Prop_HighRpoS_lowrrn
Influence graph
Signal
TopA
RpoS
Atomic proposition low_rrn
Value
FocalSet
Derivatives
StateDescriptors
Crp
z_Crp t_Crp_1 ... ... t_Crp_2 t_Crp_3 ... ... max_Crp
Cya
z_Cya t_Cya_1 k_Cya_1/g_Cya t_Cya_2 t_Cya_3 ... max_Cya
Fis
z_Fis t_Fis_1 k_Fis_1/g_Fis t_Fis_2 t_Fis_3 t_Fis_4 t_Fis_5 ... max_Fis
GyrAB
z_GyrAB t_GyrAB_1 t_GyrAB_2 k_GyrAB/g_GyrAB max_GyrAB
GyrI
z_GyrI t_GyrI_1 t_GyrI_2 k_GyrI/g_GyrI max_GyrI
RpoS
z_RpoS k_RpoS/(g_RpoS_1+g_RpoS_2) t_RpoS k_RpoS/g_RpoS_1 max_RpoS
RssB
z_RssB t_RssB k_RssB_1/g_RssB ... max_RssB
Signal
z_Signal t_Signal max_Signal
TopA
z_TopA t_TopA_1 t_TopA_2 ... k_TopA_2/g_TopA ... max_TopA
rrn
z_rrn k_rrn_2/g_rrn t_rrn (k_rrn_2+k_rrn_1)/g_rrn max_rrn
Help
