# Supplementary material for: A service-oriented architecture for integrating the modeling and formal verification of genetic regulatory networks
Source: BMC Bioinformatics. 2009 Dec 30;10:450. doi: 10.1186/1471-2105-10-450 (PMC2813247; doi:10.1186/1471-2105-10-450)
Supplement: Additional file 3 — Verification options window. Configuration of a verification request by specifying the model checker plugin to be used and, if the plugin supports an implicit representation of the FSTS, the initial conditions for the qualitative simulation of the network. [file 1471-2105-10-450-S3.PDF]

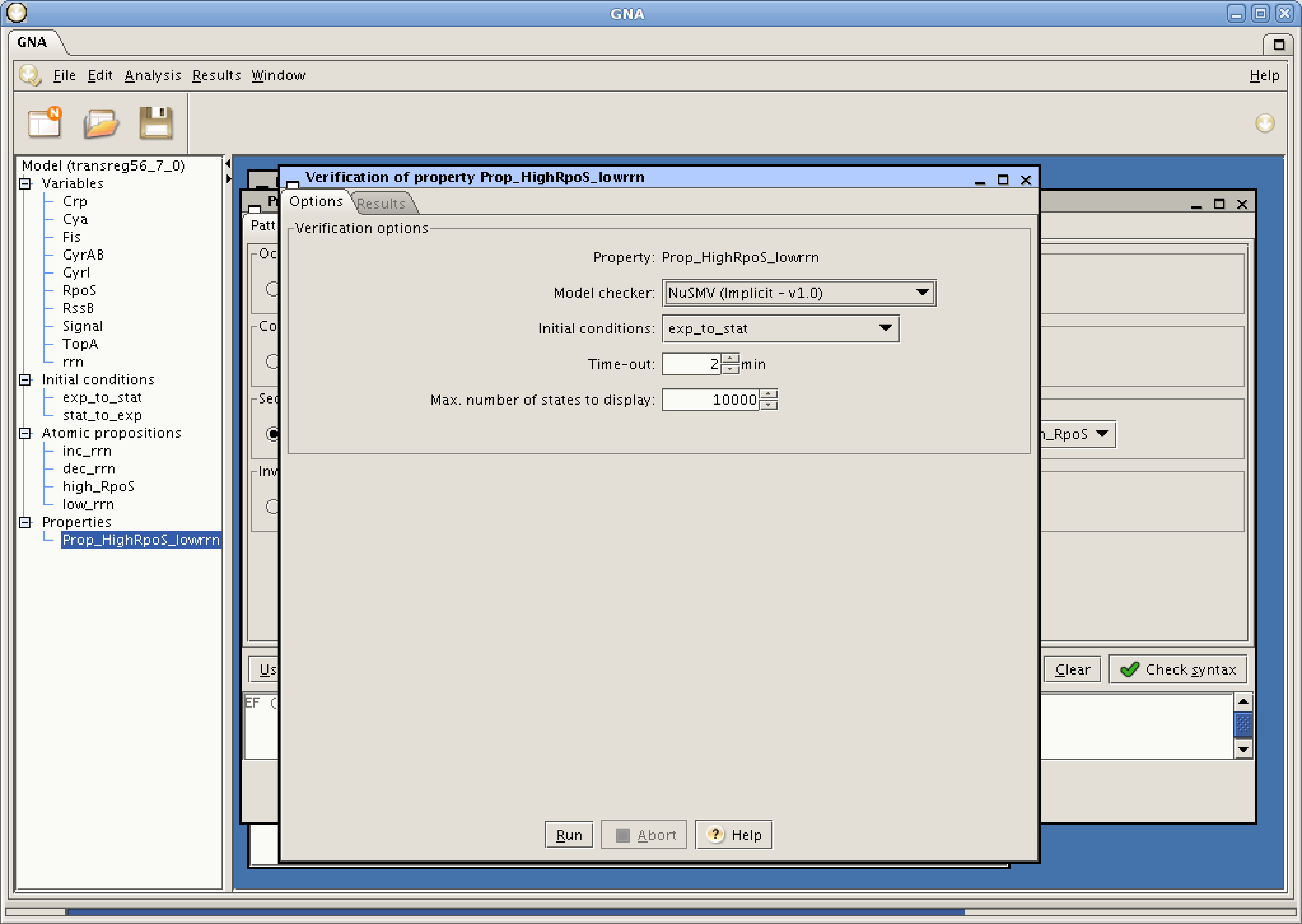
GNA
GNA
File Edit Analysis Results Window
Help
Model (transreg56_7_0)
Variables
Crp
Cya
Fis
GyrAB
GyrI
RpoS
RssB
Signal
TopA
rrn
Initial conditions
exp_to_stat
stat_to_exp
Atomic propositions
inc_rrn
dec_rrn
high_RpoS
low_rrn
Properties
Prop_HighRpoS_lowrrn
Verification of property Prop_HighRpoS_lowrrn
Options
Results
Verification options
Property: Prop_HighRpoS_lowrrn
Model checker: NuSMV (Implicit - v1.0)
Initial conditions: exp_to_stat
Time-out: 2 min
Max. number of states to display: 10000
Run
Abort
Help
Clear
Check syntax
