# Supplementary material for: A service-oriented architecture for integrating the modeling and formal verification of genetic regulatory networks
Source: BMC Bioinformatics. 2009 Dec 30;10:450. doi: 10.1186/1471-2105-10-450 (PMC2813247; doi:10.1186/1471-2105-10-450)
Supplement: Additional file 4 — Verification result. Results of the verification of a complex biological property, composed of a verdict (true) and the corresponding witness. The latter consists of a sequence of states containing a cycle (see left panel). The value of the concentration of the variables in the selected states is shown, presenting an oscillation of the concentration of the variable Fis (see right panel). [file 1471-2105-10-450-S4.PDF]

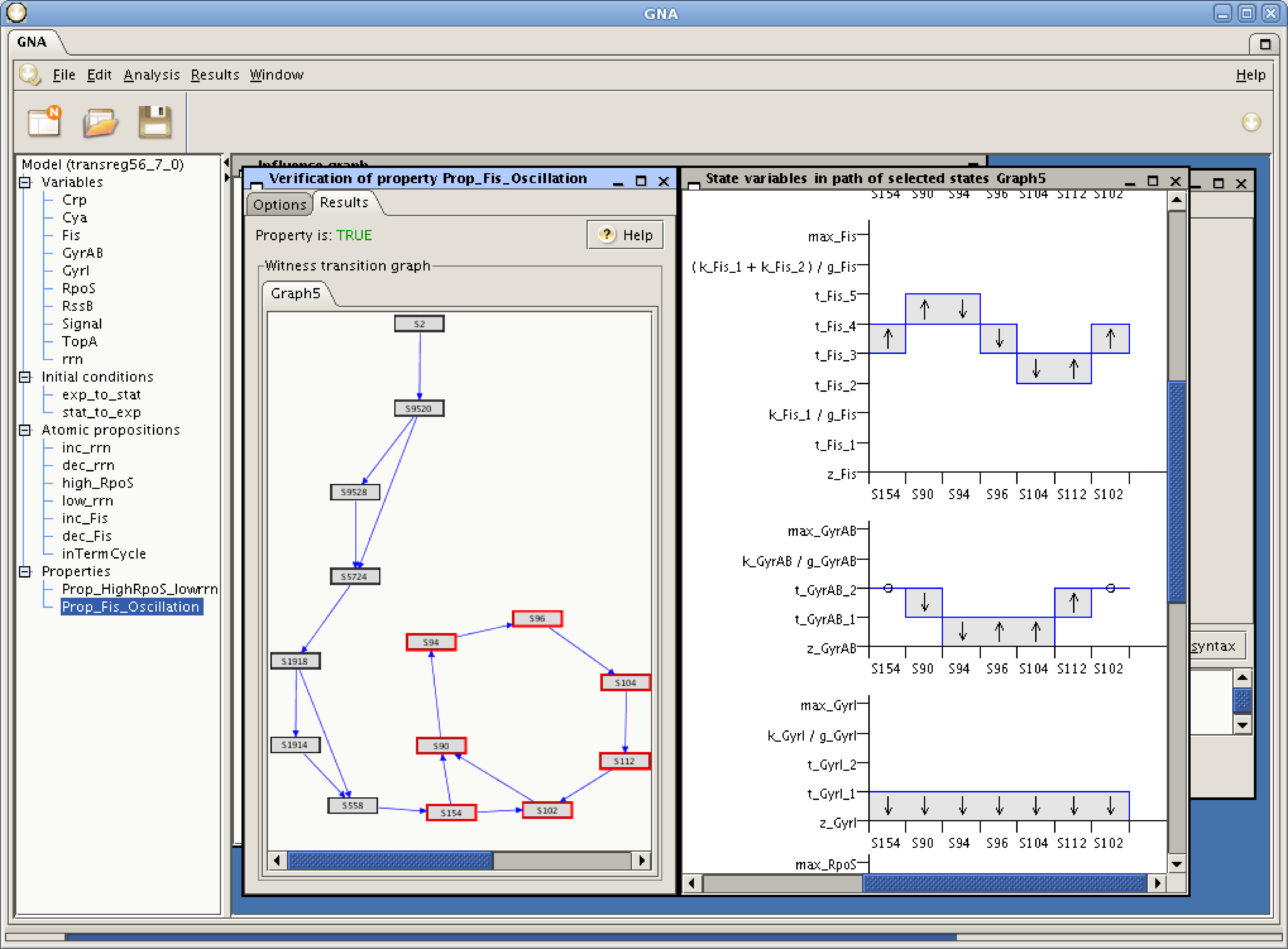
GNA
File Edit Analysis Results Window
Help
Model (transreg56_7_0)
Variables
Crp
Cya
Fis
GyrAB
GyrI
RpoS
RssB
Signal
TopA
rrn
Initial conditions
exp_to_stat
stat_to_exp
Atomic propositions
inc_rrn
dec_rrn
high_RpoS
low_rrn
inc_Fis
dec_Fis
inTermCycle
Properties
Prop_HighRpoS_lowrrn
Prop_Fis_Oscillation
Verification of property Prop_Fis_Oscillation
Options
Results
Property is: TRUE
Help
Witness transition graph
Graph5
S2
S9520
S9528
S5724
S1918
S1914
S558
S154
S102
S90
S94
S96
S104
S112
State variables in path of selected states Graph5
max_Fis
(k_Fis_1 + k_Fis_2) / g_Fis
t_Fis_5
t_Fis_4
t_Fis_3
t_Fis_2
k_Fis_1 / g_Fis
t_Fis_1
z_Fis
S154 S90 S94 S96 S104 S112 S102
max_GyrAB
k_GyrAB / g_GyrAB
t_GyrAB_2
t_GyrAB_1
z_GyrAB
S154 S90 S94 S96 S104 S112 S102
max_GyrI
k_GyrI / g_GyrI
t_GyrI_2
t_GyrI_1
z_GyrI
S154 S90 S94 S96 S104 S112 S102
max_RpoS
syntax
